# Non-canonical regulation of glutathione and trehalose biosynthesis characterizes non-*Saccharomyces* wine yeasts with poor performance in active dry yeast production

**DOI:** 10.15698/mic2018.04.624

**Published:** 2018-01-26

**Authors:** Esther Gamero-Sandemetrio, Lucía Payá-Tormo, Rocío Gómez-Pastor, Agustín Aranda, Emilia Matallana

**Affiliations:** 1 Department of Biotechnology, Institute for Agrochemistry and Food Technology, CSIC, Valencia, Spain.; 2 Institute for Integrative Systems Biology I2SysBio, Universitat de València/CSIC, Valencia. Spain.; 3 Present address: Department of Neuroscience, Medical School, University of Minnesota, Minneapolis, Minnesota, USA.

**Keywords:** non-Saccharomyces yeasts, active dry wine yeasts, food-grade argan oil, oxidative damage, antioxidant defense

## Abstract

Several yeast species, belonging to *Saccharomyces* and non-*Saccharomyces* genera, play fundamental roles during spontaneous must grape fermentation, and recent studies have shown that mixed fermentations, co-inoculated with *S. cerevisiae* and non-*Saccharomyces* strains, can improve wine organoleptic properties. During active dry yeast (ADY) production, antioxidant systems play an essential role in yeast survival and vitality as both biomass propagation and dehydration cause cellular oxidative stress and negatively affect technological performance. Mechanisms for adaptation and resistance to desiccation have been described for *S. cerevisiae,* but no data are available on the physiology and oxidative stress response of non-*Saccharomyces* wine yeasts and their potential impact on ADY production. In this study we analyzed the oxidative stress response in several non-*Saccharomyces* yeast species by measuring the activity of reactive oxygen species (ROS) scavenging enzymes, e.g., catalase and glutathione reductase, accumulation of protective metabolites, e.g., trehalose and reduced glutathione (GSH), and lipid and protein oxidation levels. Our data suggest that non-canonical regulation of glutathione and trehalose biosynthesis could cause poor fermentative performance after ADY production, as it corroborates the corrective effect of antioxidant treatments, during biomass propagation, with both pure chemicals and food-grade argan oil.

## INTRODUCTION

Grape must fermentation is a complex biochemical process in which diverse yeasts, including *Saccharomyces* and non-*Saccharomyces* species, play fundamental roles in transforming grape sugars into ethanol, carbon dioxide, and hundreds of other secondary products. Early fermentation stages are dominated by non-*Saccharomyces* yeasts that are gradually replaced with the species *Saccharomyces cerevisiae*, which takes over fermentation 1. The dominance of non-*Saccharomyces* yeasts in early fermentation stages has a major impact on the aromatic composition and sensory properties of wine 2,3,4. Consequently, many researchers have investigated the metabolic properties of various non-*Saccharomyces* yeast species and their potential applications in the wine industry 5,6 to thus determine a positive impact on the body and organoleptic quality of wines. Active dry yeast (ADY) is commonly used as an inoculum in wine fermentations, which leads to greater process control and consistent quality 7. Recent studies have shown that mixed fermentations co-inoculated with *S.* *cerevisiae* and non-*Saccharomyces* strains can improve the analytical and aromatic profile of wines through metabolic interactions between different yeast species 3,4. However, non-*Saccharomyces* ADY usually displays poor fermentative capacity, and the production process in molasses medium gives low biomass yields. Both biomass propagation and dehydration in industrial ADY production of *S. cerevisiae* strains have been reported to cause cellular oxidative stress, and then negatively affect performance 8,9,10. Therefore, antioxidant systems play an essential role in tolerance to drying. In *S. cerevisiae,* adaptation and resistance to desiccation include protection against oxidative stress through ROS scavenging enzymes, such as catalase and glutathione reductase, and protective metabolites, such as trehalose and reduced glutathione (GSH). However, the oxidative stress response in non-*Saccharomyces* species and its putative relevance for their low yield and fermentative efficiency after ADY production have not been studied. We recently identified a set of biochemical parameters (levels of oxidized glutathione and trehalose, and catalase and glutathione reductase activities), analyzed after dehydration, which allowed the prediction of physiologically relevant phenotypes for wine *S. cerevisiae* strains 11, and we demonstrated that a low level of oxidative defense characterizes deficiently performing strains. So the study of these parameters can help to define the antioxidant response of non-*Saccharomyces* strains, and to also find correlations with their resistance and performance during ADY production. Due to the interest of these yeast species as mixed starters with *S. cerevisiae* strains, the design of technologically affordable treatments for improving their performance as ADY would have important biotechnological implications for wine making 12,13. Recently, we proposed using three pure antioxidant molecules (ascorbic, caffeic and oleic acids), and argan oil as a food-grade natural antioxidant, in industrial processes that involve high cellular oxidative stress, such as the biotechnological production of dry starters 14. L-ascorbic acid (vitamin C) acts as a reducing substrate for peroxidases 15. *S. cerevisiae* strains synthesize the analog, erythroascorbate, which prevents apoptosis induced by pro-oxidants, increases the levels of GSH and lowers ROS levels 16. Antibacterial, antiviral, anti-inflammatory, anticancer and antioxidant activities have been described for caffeic acid in several organisms 17,18,19,20. At low doses, it suppresses lipid peroxidation 17 and blocks ROS 19. Under exogenous oxidative stress, caffeic acid increases GSH levels and lowers ROS levels in* S. cerevisiae* 16. Oleic acid supplementation in* S. cerevisiae* growth media can alleviate oxidative stress during must fermentation 21 as the lipid composition of the cell membrane modulates the activity of enzymes and membrane-associated transporter functions 22,23. Finally, argan oil is an example of a natural product rich in antioxidants, which is now commercialized in both cosmetic and food grades, and displays antiproliferative, antidiabetic and cardiovascular risk-preventive effects 24,25.

This study aimed to dissect the oxidative defense properties of several non-*Saccharomyces* wine yeast species during ADY production by determining the previously selected biomarkers and studying the protective antioxidant effects of pure chemicals and natural products. To do this, non-*Saccharomyces* yeasts were propagated on molasses supplemented, or not, with the aforementioned antioxidants, and were then dehydrated. The resulting ADY products were assayed for fermentative performance, biomass yield, the above-mentioned biomarkers, and also for effects on wine produced in mixed fermentations.

## RESULTS

### Deficient oxidative defenses in non-*Saccharomyces* species cause inappropriate fermentative performance for active dry wine yeast production

Five non-*Saccharomyces* wine yeast strains were selected according to their physiology and potential contribution to wine organoleptic properties, and were tested for performance during and after ADY production by assaying biomass yield and fermentation capacity in comparison to the efficient commercial *S. cerevisiae* wine strain T73 (Table 1). In general, non-*Saccharomyces* yeasts give low biomass yields, except for *C. stellata* whose performance was almost 50% higher than the control strain T73. Similar results were obtained for fermentative capacity, but in this case *T. delbrueckii* was the species that displayed better fermentative capacity, similarly to the control strain. In order to gain further information on the molecular causes of the physiological behavior of these wine yeasts, oxidative macromolecular damage markers were analyzed. As seen in Table 1, lipid peroxidation and protein carbonylation were not significantly higher for any of the five non-*Saccharomyces* species compared to the control strain, even both parameters were lower for most of them. *H. osmophila* proved similar to the control strain in lipid peroxidation terms, whereas *T. delbrueckii* did in protein carbonylation terms.

**Table 1:** Biomass yield, fermentative capacity and oxidatively damaged macromolecules in T73 (*S. cerevisiae*) and non-*Saccharomyces* wine yeast strains. ^a^ Cell growth after 24 h in the molasses medium at 30°C, measured as OD_600_. ^b^ Fermentative capacity from ADY; measured in YPGF medium for 6 h at 30^°^C. ^c^ Lipid peroxidation and protein carbonylation from ADY. Protein carbonyl was expressed as Ci/ Pi, where Ci is the protein carbonyl content quantified by an image analysis and Pi is total protein from coomassie-stained membranes. *SD of three independent experiments in brackets.

**Strain**	**Biomass yield^a^**	**Fermentative capacity (mL CO_2_/ 10^7^ cells)^b^**	**Lipid peroxidation (pmol MDA/ mg protein)^c^**	**Protein carbonylation (Ci/Pi)^c^**
**T73**	18.42 (±0.28)	10.52 (± 0.30)	26.85 (±2.04)	23.26 (±1.38)
***C. stellata***	25.84 (±0.39)	1.77 (±0.45)	17.16 (±1.00)	15.20 (±1.10)
***T. delbrueckii***	10.03 (±0.79)	11.33 (±0.94)	19.61 (±0.57)	24.93 (±0.51)
***P. fermentans***	8.73 (±0.19)	1.23 (±0.52)	17.73 (±0.84)	14.31 (±0.57)
***H. osmophila***	10.17 (±1.09)	3.81 (±0.50)	24.80 (±1.86)	17.88 (±0.26)
***H. guilliermondii***	9.2 (±2.10)	2.81 (±0.62)	20.82 (±1.57)	12.99 (±0.70)

As the analyzed markers of lipid and protein oxidative damage did not provide clues about the molecular basis of non-*Saccharomyces* yeasts’ suboptimal performance, we extended the study to other biochemical parameters, such as glutathione and trehalose levels, and the enzymatic activities of catalase and glutathione reductase (Table 2). In general, all the non-*Saccharomyces* yeasts displayed a low GSH/GSSG ratio compared to strain T73 in fresh cells (Table 2), which is a negative factor for a healthy cellular redox state 26. These data suggest that regulation of glutathione biosynthesis in non-*Saccharomyces* wine yeasts might be a limiting factor for adaptation during biomass propagation compared to *S. cerevisiae* strains. This general difference is not maintained after dehydration and only two of the *non-Saccharomyces* species displayed very low GSH/GSSG ratios in dry cells.

**Table 2:** Biomarkers of the redox state in ADY from T73 (*S. cerevisiae*) and non-*Saccharomyces* wine yeast strains. *SD of three independent experiments in brackets. GR (Glutathione reductase); CAT (Catalase).

**Strain**	**GSH/GSSG (nmol/mg cell)**		**Trehalose (µg/mg cells)**		**GR (dry/fresh)**	**CAT (dry/fresh)**
	**Fresh cells**	**Dry cells**	**Fresh cells**	**Dry cells**
**T73**	98.18 (±5.12)	14.24 (±1.12)	85.24 (±0.79)	149.57 (±0.12)	0.61 (±0.03)	3.76 (±0.09)
***C. stellata***	31.65 (±5.24)	16.87 (±8.56)	41.43 (±2.13)	14.02 (±0.15)	0.40 (±0.05)	1.4 (±0.06)
***T. delbrueckii***	37.56 (±5.78)	1.99 (±1.27)	7.21 (±0.55)	80.33 (±7.79)	2.57 (±0.03)	0.26 (±0.03)
***P. fermentans***	37.59 (±4.51)	12.47 (±3.74)	2.33 (±0.08)	37.35 (±0.59)	0.43 (±0.01)	1.57 (±0.09)
***H. osmophila***	75.41 (±10.4)	6.95 (±2.77)	5.14 (±0.41)	17.67 (±2.07)	3.30 (±0.04)	2.13 (±0.04)
***H. guilliermondii***	23.02 (±5.79)	13.75 (±1.97)	3.67 (±0.08)	8.53 (±097)	1.37 (±0.05)	3.98 (±0.06)

Regarding intracellular trehalose accumulation (Table 2), all the strains, except for *C. stellata,* showed that this protective metabolite accumulated after dehydration, as observed for the control strain. It is noteworthy that *C. stellata *displayed the lowest fermentative capacity in YPGF medium after rehydration*.* Although trehalose accumulation was induced by desiccation, these non-*Saccharomyces* species generally possess low levels of trehalose compared to the T73 control strain, which could be related to their generally poor performance during ADY production, where *T. delbrueckii* was the only exception, and also the strain that displayed the highest fermentative capacity (Table 1).

Regarding antioxidant enzymes, Table 2 shows the variation in the levels of glutathione reductase (GR) and catalase activities in dry versus fresh cells. In general terms, the desiccation-induced changes in both enzymatic activities did not provide a good correlation with the physiological parameters. For GR activity in the different non-*Saccharomyces* strains, it did not correlate with the GSH/GSSG ratios (Table 1), which could reinforce the hypothesis of the deficient, or different, regulation of glutathione metabolism in these yeast species. For catalase, although no large differences in the ratios between activity in dry and fresh cells were observed, it is worth stressing the high values of activity detected in both states for *P. fermentans* (fresh 30.77 and dry 48.39 U/mg protein; not shown), and specially for *C. stellata* (fresh 159.2 and dry 223.68 U/mg protein), compared to strain T73 (fresh 8.44 and dry 31.72 U/mg protein). The very marked catalase activity in fresh cells and the further induction after dehydration in* C. stellata* and *P. fermentans *could be related to their low fermentative capacity (Table 1). *T. delbrueckii* had moderate catalase activity in fresh cells (13.27 U/mg protein), which even reduced (3.49 U/mg protein) after dehydration. This suggests lower oxidative stress compared to the other strains, which could be consistent with its high fermentative capacity*.*

### Enhancement of the oxidative stress response by antioxidants improves biomass yield but not fermentative efficiency

Three pure antioxidant molecules (ascorbic acid, caffeic acid and oleic acid), previously selected for their ability to improve fermentative performance in *S. cerevisiae* wine strains 14, were supplemented in molasses medium during biomass propagation to further investigate the relevance of the oxidative stress response and adaptation in these non-*Saccharomyces* wine yeasts, and to check their potential protective effects on fermentative performance in winemaking.

The individual effects of each antioxidant molecule on the physiological and biochemical parameters for the five strains under study and of the control T73 strain are shown in Table 3 where the value of each parameter is shown in relation to the data when antioxidants are absent for dry cells in Tables 1 and 2, except for biomass yield which is always given only in fresh cells. The three antioxidants generally increased biomass yields, with similar values for all the non-*Saccharomyces,* and also for the control *S. cerevisiae* strain T73. As for their action on fermentative capacity, the strongest positive effect was observed with the three antioxidants for* C. stellata* (3.7-fold in ascorbic acid; 3-fold in caffeic acid; 5-fold in oleic acid) and *H. osmophila* (3-fold in ascorbic acid; 3.1-fold in caffeic acid; 4-fold in oleic acid). Other strain or antioxidant-dependent effects were also observed as the positive effect of oleic acid of the fermentative capacity of *T. delbrueckii* (1.91-fold).

**Table 3:** Effect of ascorbic acid (first line), caffeic acid (second line) or oleic acid (third line) supplementations on yeast performance and oxidative response. Data relative to those in Tables 1 and 2. *SD of three independent experiments in brackets.

	***T73***	***C. stellata***	***T. delbrueckii***	***P. fermentans***	***H. osmophila***	***H. guilliermondii***
**Biomass yield**	1.16 ± 0.03	0.99 ± 0.07	2.83 ± 0.08	2.11 ± 0.01	1.14 ± 0.08	2.64 ± 0.01
	1.54 ± 0.01	1.22 ± 0.03	2.06 ± 0.09	2.37 ± 0.03	2.99 ± 0.05	2.79 ± 0.01
	1.81 ± 0.02	1.38 ± 0.02	2.81 ± 0.05	3.22 ± 0.01	2.46 ± 0.02	3.33 ± 0.05
**Fermentative Capacity**	2.11 ± 0.01	3.7 ± 0.05	0.96 ± 0.08	1.00 ± 0.05	3.04 ± 0.08	1.66 ± 0.06
	0.86 ± 0.02	3.06 ± 0.01	1.07 ± 0.01	1.08 ± 0.01	3.14 ± 0.07	1.61± 0.05
	0.86 ± 0.01	5.03 ± 0.02	1.91 ± 0.01	0.58 ± 0.01	4.08 ± 0.03	1.88 ± 0.01
**Lipid Peroxidation**	0.87 ± 0.05	0.93 ± 0.04	0.69 ± 0.01	0.55 ± 0.01	0.79 ± 0.03	0.39 ± 0.01
	0.79 ± 0.02	0.88 ± 0.02	1.06 ± 0.01	1.07 ± 0.03	0.95 ± 0.02	1.02 ± 0.01
	1.08 ± 0.01	0.82 ± 0.02	0.69 ± 0.01	0.65 ± 0.01	0.76 ± 0.02	0.79 ± 0.03
**Protein Carbonylation**	1.04 ± 0.05	1.05 ± 0.07	0.54 ± 0.04	1.27 ± 0.06	1.44 ± 0.07	1.55 ± 0.09
	1.16 ± 0.01	1.19 ± 0.01	0.81 ± 0.02	1.33 ± 0.03	1.29 ± 0.01	1.02 ± 0.01
	1.03 ± 0.02	1.04 ± 0.01	0.71 ± 0.01	0.96 ± 0.03	1.29 ± 0.05	1.47 ± 0.04
**GSH/GSSG**	1.13 ± 0.01	1.2 ± 0.02	1.19 ± 0.01	1.28 ± 0.01	1.13 ± 0.01	1.65 ± 0.02
	1.24 ± 0.02	2.0 ± 0.02	1.01 ± 0.01	1.33 ± 0.05	0.85 ± 0.02	1.44 ± 0.01
	1.38 ± 0.02	1.57 ± 0.04	0.93 ± 0.01	1.47 ± 0.01	0.90 ± 0.02	1.66± 0.01
**Trehalose**	6.28 ± 0.21	65.08 ± 0.25	3.31 ± 0.01	0.35 ± 0.11	0.07 ± 0.01	0.30 ± 0.01
	3.66 ± 0.02	54.86 ± 2.11	2.00 ± 0.01	0.46 ± 0.01	2.76 ± 0.05	2.83 ± 0.02
	4.34 ± 0.02	44.6 ± 1.01	2.21 ± 0.01	0.93 ± 0.01	1.80 ± 0.04	4.18 ± 0.06
**GR_ADY_/GR_BIOMASS_**	1.77 ± 0.02	6.82 ± 0.04	0.92 ± 0.02	2.97 ± 0.01	0.26 ± 0.01	0.39 ± 0.01
	2.68 ± 0.05	5.35 ± 0.06	1.47 ± 0.04	8.65 ± 0.05	0.57 ± 0.01	0.13 ± 0.02
	3.82 ± 0.05	5.97 ± 0.06	1.50 ± 0.02	2.86 ± 0.03	0.37 ± 0.01	0.39 ± 0.01
**CAT_ADY_/ CAT_BIOMASS_**	0.33 ± 0.04	2.08 ± 0.08	1.80 ± 0.0	0.86 ± 0.07	0.25 ± 0.02	0.18 ± 0.09
	0.26 ± 0.01	1.64 ± 0.03	1.03 ± 002	0.54 ± 0.01	0.33 ± 0.01	0.48 ± 0.02
	0.54 ± 0.02	3.56 ± 0.05	1.16 ± 0.02	1.94 ± 0.02	0.32 ± 0.01	0.01 ± 0.01

Lipid peroxidation was slightly diminished by treatment with any of the antioxidants in all the non-*Saccharomyces* species, and also in the T73 control strain, except for oleic acid which did not reduce this molecular damage in the *S. cerevisiae* strain.

Once again, the antioxidants effects of protein carbonylation were not homogeneous, and both antioxidant and strain-dependent behaviors were observed. However, the level of this oxidative damage to proteins slightly increased with the antioxidant treatments in four of the five non-*Saccharomyces* yeasts, these being *C. stellata,* *P. fermentans,* and both *Hanseniaspora* species. All the antioxidants diminished protein carbonylation in *T. delbrueckii.*

The effect on the ratio between reduced and oxidized glutathione was similar for all three antioxidants, and for all the analyzed yeasts. A slightly increase in the GSH/GSSG ratio was observed, which suggests that the protective effect improves the redox cellular state by elevating the GSH level and/or diminishing GSSG levels and, therefore, also oxidative stress.

The effects of ascorbic, caffeic and oleic acids on trehalose levels indicated two extreme responses in the two worse performing species in fermentative capacity terms: those of *C. stellata* and *P. fermentans*. *C. stellata* displayed an anomalous reduction in trehalose levels after dehydration (Table 2), which was reversed by the treatment with antioxidants (Table 3), which could explain the increased fermentative capacity produced in this yeast by the three compounds. *P. fermentans*, however, showed significantly increased trehalose accumulation during dehydration (Table 2), and the antioxidant treatments diminished it (Table 3), with very little effect on fermentative capacity. The trehalose accumulation of the two *Hanseniaspora* species, also characterized by their low fermentative capacity, was affected differentially by the three antioxidants, and no correlation was found between the trehalose level and the effect on fermentative capacity.

Finally, GR activity after dehydration was increased by all the treatments for the two non-*Saccharomyces *yeasts which displayed poor induction by dehydration (see Table 2), with different effects noted on the fermentative capacity of *C. stellata* and *P. fermentans, *where the former improved, but not the latter. In the two *Hanseniaspora* species, the three antioxidants lowered the GR activity ratio between dry and fresh cells, which was relatively high in their absence (Table 2). The mildest effect of the three antioxidants on catalase activity was given for *T. delbrueckii*, the only species that showed lower activity after dehydration (Table 2), whereas all the other yeasts, except for *C. stellata*, lowered the ratio of the activity between dry and fresh cells under antioxidant treatment. Once again, no clear correlation with fermentative capacity was found for any individual antioxidant treatment.

### Argan oil supplementation mimics the beneficial effects and the biomarkers patterns of individual antioxidant treatments

Argan oil was selected as a natural compound given its high content in ascorbic, caffeic and oleic acids, and because it improves both biomass yield and fermentative performance in ADY for *S. cerevisiae* wine strains 14.

**Figure 1:**
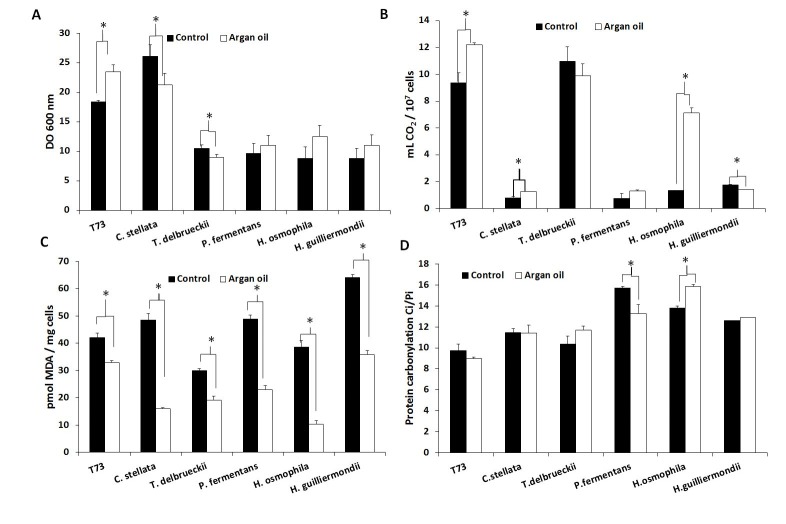
FIGURE 1: Effects of argan oil supplementation on physiological performance and oxidative damage. **(A)** Biomass yield, measured as OD at 600 nm. **(B)** Fermentative capacity measured as the volume of CO_2_ produced per 10^7^ dry cells. **(C)** Lipid peroxidation in dry cells was expressed as the amount of MDA per mg of cells. **(D)** Protein carbonyl in dry cells was expressed as Ci/ Pi, where Ci is the protein carbonyl content quantified by an image analysis and Pi is the total protein from coomassie-stained membranes. Error bars correspond to the SD value of three independent experiments. (*) significantly differed from the control (non-supplemented molasses) with *p* < 0.05.

Supplementation of molasses with 6 mg/mL of argan oil for biomass propagation increased the fermentative capacity in ADY for the control strain T73 and *C. stellata*, and especially for *H. osmophila* (Figure 1B). However, biomass yield clearly improved only in strain T73 (Figure 1A). Unlike the individual antioxidant treatments, argan oil supplementation generally protected lipid peroxidation (Figure 1C), with *H. osmophila *being the species for which this protection was greater. Argan oil supplementation did not significantly affect protein carbonylation (Figure 1D), but for *P. fermentans* and *H. osmophila*, contrary effects were observed, and no correlation was found with biomass yield or fermentative capacity.

**Figure 2:**
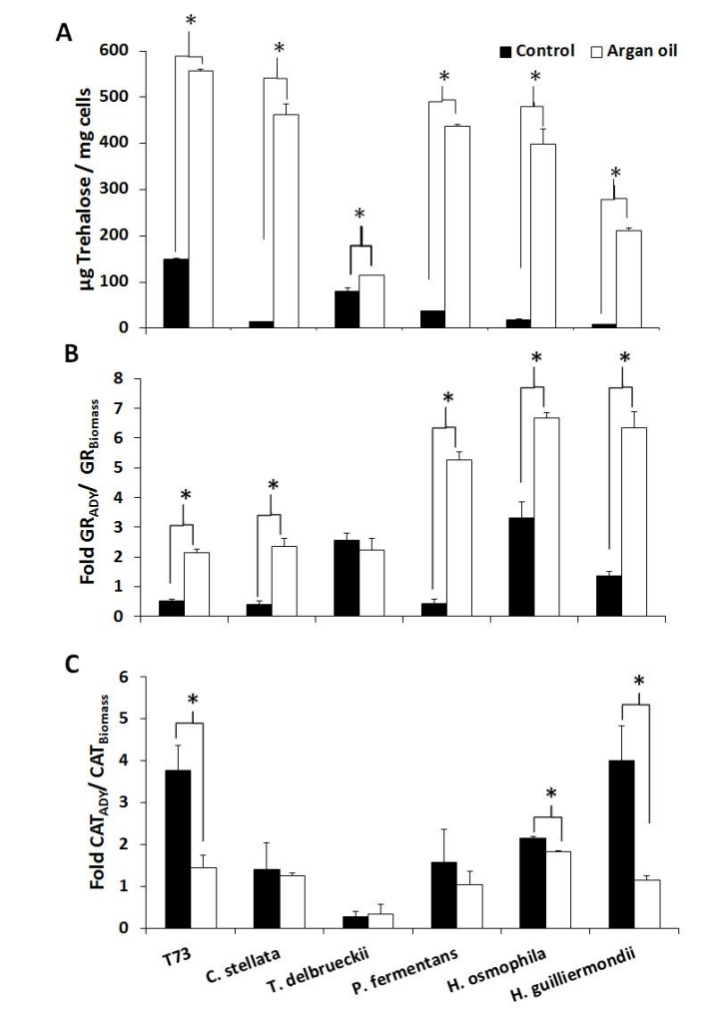
FIGURE 2: Analysis of the predictive biomarkers in ADY after argan oil supplementation during biomass propagation. **(A)** Trehalose content after drying. **(B)** Increment in glutathione reductase (GR) activity after drying. **(C)** Increment in catalase activity after drying. Error bars correspond to the SD of three independent experiments. (*) significantly differed from the control with a *p* < 0.05.

**Figure 3:**
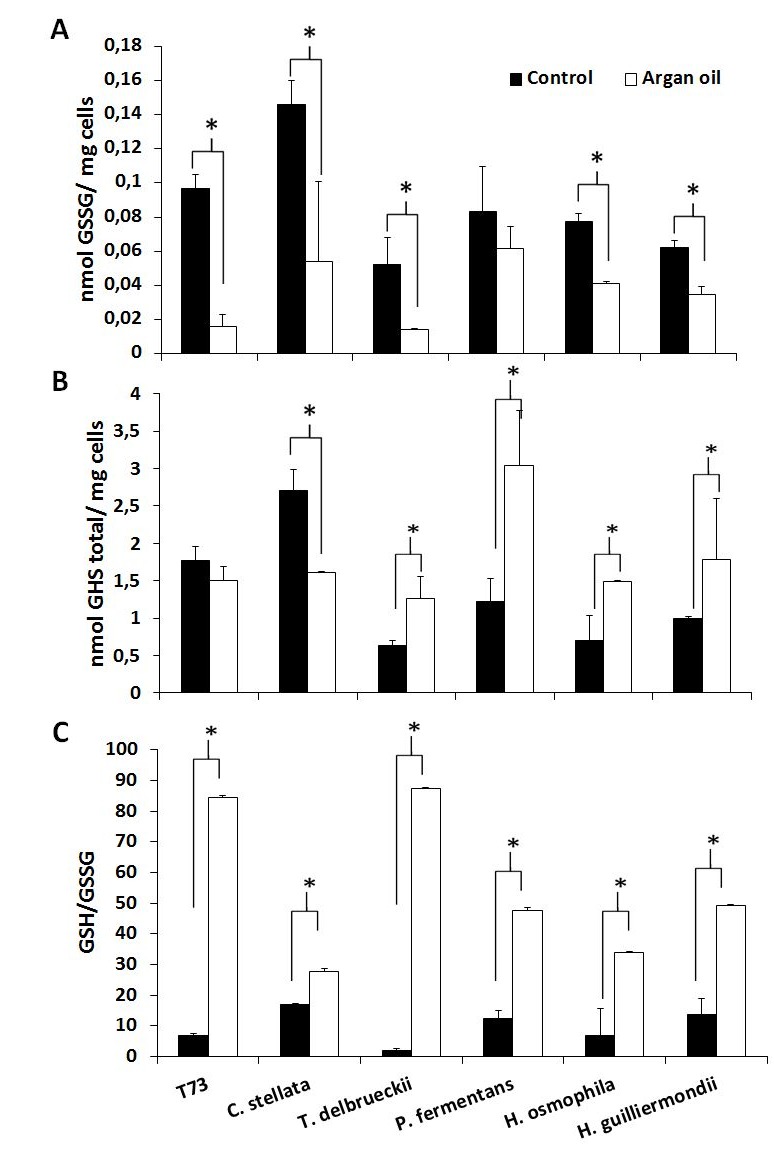
FIGURE 3: Analysis of the glutathione levels in ADY after argan oil supplementation during biomass propagation. **(A)** Oxidized glutathione after drying. **(B)** Total glutathione after drying. **(C)** The GSH/GSSG ratio after drying. Error bars correspond to the SD of three independent experiments. (*) significantly differed from the control with a *p* < 0.05.

The aforementioned predictive biomarkers were also analyzed for the ADY obtained with argan oil supplementation (Figures 2 and 3). Overall, the trehalose levels (Figure 2A) increased for all the strains and the GSSG levels lowered (Figure 3A). According to the data of both total glutathione and the GSH/GSSG ratio (Figure 3B and 3C), argan oil would stimulate glutathione synthesis in all the non-*Saccharomyces* yeasts, except for *C. stellata*. Greater induction of GR activity (Figure 2B) was also observed, except for *T. delbrueckii*, which also had the lowest GSSG levels. As for the effects on catalase activity (Figure 2C), argan oil treatment only lowered catalase induction after desiccation in the *Hanseniaspora* species, and also in the control T73 strain.

**Figure 4:**
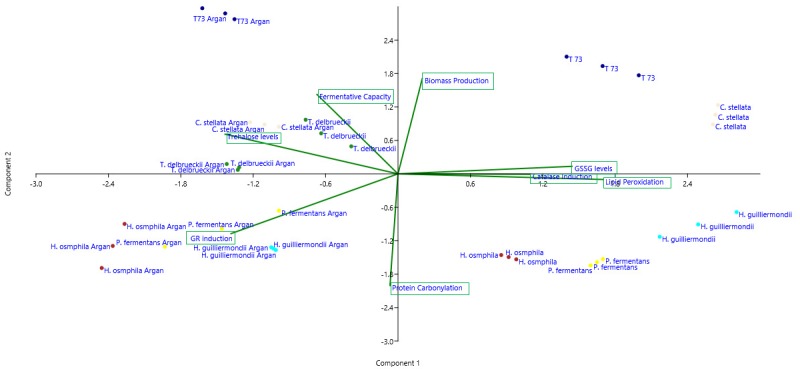
FIGURE 4: Principal components (PCA) statistical analysis of the argan oil effects on the physiological and biochemical biomarkers with represented total variance of 79%. Component 1 reflects 39.55% total variance (with a positive correlation with biomass yield, trehalose levels and glutathione reductase activity) and Component 2 reflects 30.45% total variance (with a positive correlation with GSSG levels). Lines belong to the variance of the dependent variables or the biochemical biomarkers measured (biomass yield, fermentative capacity, lipid peroxidation, protein carbonylation, protective metabolites and enzymatic activities) arranged in two dimensions according to Components 1 and 2. Study strains and conditions (control and argan oil supplementation) are labeled with different symbols: T73 (dark blue); *C. stellata* (pink); *T. delbrueckii* (green); *P. fermentans* (yellow); *H. osmophila* (red); and *H. guilliermondii* (blue), and are associated with the dependent variable, which differs from the other strains and conditions.

Given the difficulty to simultaneously compare the effects on all the strains and parameters, a statistical principal component analysis (PCA) was performed and the 2-dimensional graph (2D-plot) was used to better define the effect of argan oil supplementation on the physiological and oxidative stress parameters (Figure 4). As we can see, the argan oil-treated samples appear on the left of the graph and directly correlate with further increases, compared to the control situation, of the following parameters: induction of GR activity, fermentative capacity, trehalose levels and biomass production. An inverse correlation can also be seen with these parameters: induction of catalase activity, GSSG levels and lipid peroxidation to fermentative capacity. More specifically, we can identify the main effect exerted by argan oil supplementation on each strain, where: the *P. fermentans *and *Hanseniaspora* species would further increase their GR activity; the trehalose levels of *C. stellata* would increase; *T. delbrueckii* would be associated with both induction of GR activity and trehalose levels. These results also correlate with the data observed in the experiments shown in Figures 1 and 2.

### Argan oil supplementation in ADY production improves *H. osmophila *viability during alcoholic fermentation of natural must

As molasses supplementation with argan oil has beneficial effects on the fermentative capacity of *H. osmophila* ADY (Figure 1B), we decided to check whether it would also influence the behavior of this ADY when used for winemaking on natural must, as this species is of much enological interest thanks to its contribution with positive flavor compounds.

**Figure 5:**
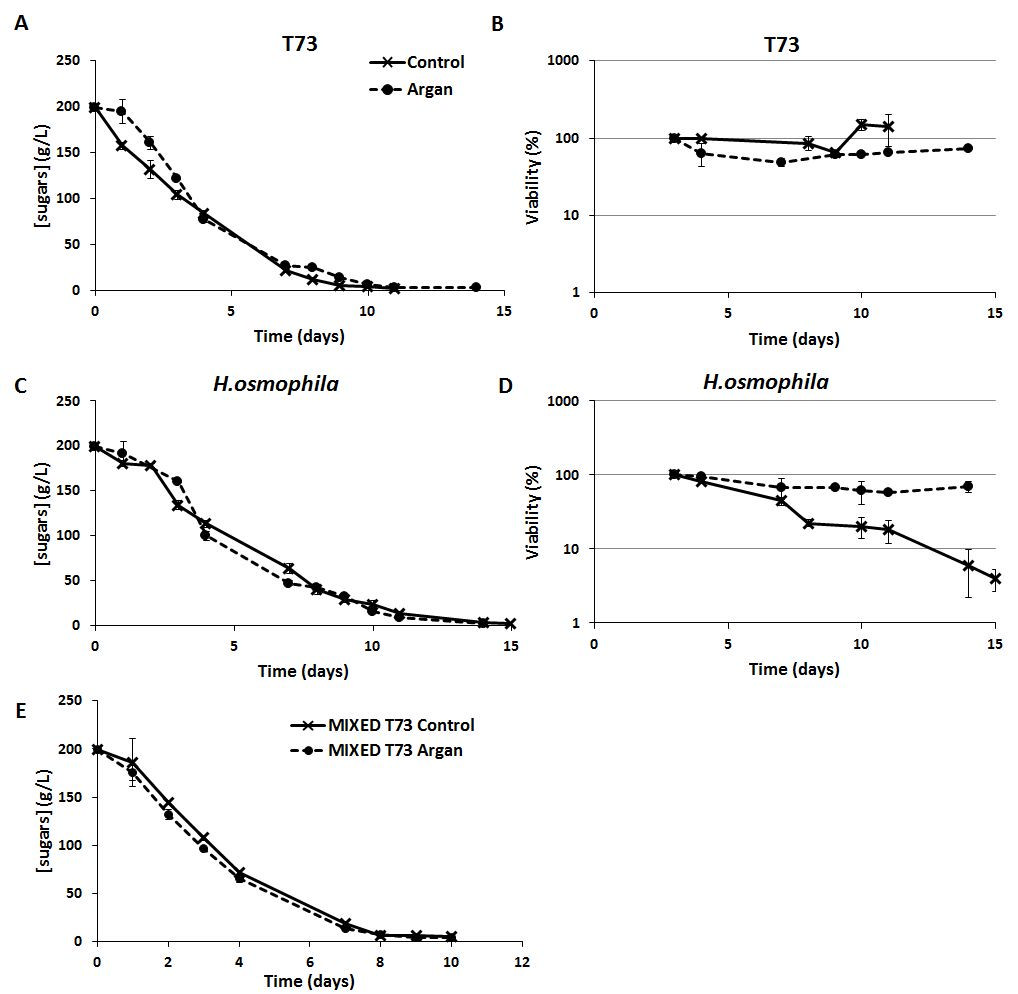
FIGURE 5: Fermentation parameters for vinifications with T73 and *H. osmophila* pure cultures and with mixed cultures. Sugar consumption **(A and C)** and viability **(B and D)** profiles during natural must fermentation inoculated with the ADY obtained from molasses in the absence (control) and presence of argan oil (argan). Panel **E** shows sugar consumption in vinifications conducted by mixed starters where *S. cerevisiae* T73 ADY was obtained from molasses in the absence (control) or presence of argan oil (argan) and the *H. osmophila* ADY was always obtained from molasses supplemented with argan oil. Fermentations were considered complete when the sugars concentration went below 2 g/L.

Figure 5 shows the sugar consumption and viability profiles for control strain T73 (Panels A and B) and *H. osmophila* (Panels C and D) during pure culture vinifications. As we can see for both species, no differences in sugar consumption are observed between the ADY obtained by growth in standard molasses and the argan oil-supplemented molasses (Figure 5, Panel A and C). However, the control* S. cerevisiae* strain T73 (Figure 5A) consumed sugars faster than non-*Saccharomyces* species (Figure 5C), as it is observed during the first days and also in time to complete sugar consumption. This observation is in agreement with literature, where lower fermentative power has been generally described for non-*Saccharomyces* yeasts 7. The viability of the T73 control strain during wine fermentation was not affected by previously propagating the biomass for ADY production in the argan oil-supplemented molasses (Figure 5B), but it was improved by 1.8-fold for *H. osmophila *(Figure 5D)*.*

### Argan oil supplementation in ADY production does not affect organoleptic and aromatic profiles in the mixed fermentation carried out by S. *cerevisiae* and *H. osmophila *strains

As mentioned in the introduction, non-*Saccharomyces* wine yeast provides greater complexity in aroma and flavor in wines, 27. In order to check the putative effects of argan oil supplementation on this advantageous property, mixed multi-starter wine fermentations were designed using *S. cerevisiae* T73 ADY, both from standard (control) and the argan oil-supplemented molasses (argan), and *H. osmophila* ADY from the argan oil-supplemented molasses.

Figure 5, Panel E, shows the sugar consumption profiles for the mixed multi-starter vinifications. Sugar consumption in the initial fermentation phases was faster in mixed multi-starter winemaking, probably because both yeast species act from the beginning and contribute to sugar degradation. Moreover, mixed fermentation was completed after 10 days, as were the T73-conducted fermentations.

Organoleptic and sensorial characterization was also carried out for the pure culture and the mixed multistarter vinifications to identify any potential positive or detrimental effects of argan oil supplementation during ADY production. The levels of ethanol, glycerol and acetic acid, and the profile of the volatile compounds (Table 4), were determined in the final wine products.

**Table 4:** Enological parameters and volatile compounds in the wines obtained by monocultures and multi-starter mixed fermentations using the ADY obtained with the standard molasses (control) and the molasses supplemented with argan oil (argan). * nd: not detected. The average error in the chromatographic measurements was less than 5% in all cases.

				**Fermentation**			
		**T73**		***H. osmophila***		**Mixed**	
		**Control**	**Argan**	**Control**	**Argan**	**Control**	**Argan**
**Enological parameters (g/L)**	**Ethanol**	10.06	11.33	10.56	10.94	11.67	12.63
	**Glycerol**	10.55	9.39	9.44	9.45	8.39	8.81
	**Acetic acid**	0.14	0.004	0.31	0.05	0.19	0.18
**Acetate esters (mg/L)**	**Ethyl acetate**	36.39	36.75	111.73	87.79	60.63	49.96
	**Isobutyl acetate**	0.05	0.05	0.05	0.08	nd*	nd*
	**Isoamyl acetate**	0.72	0.78	0.76	1.62	0.68	0.68
	**2-phenyl-ethyl**	0.25	0.43	7.71	16.36	0.340	0.22
**Higher alcohols (mg/L)**	**2-phenylethanol**	39.42	73.67	23.41	62.33	95.56	68.53
	**Isobutyl**	59.15	61.55	52.17	76.18	73.90	57.69
	**Isoamyl alcohol**	292.0	302.74	184.28	330.53	366.83	318.13
**Fatty acid esters (mg/L)**	**Ethyl caprylate**	0.280	0.27	0.02	0.26	0.09	0.07
	**Ethyl caprate**	0.03	0.05	0.12	0.43	0.06	0.05
	**Caproate**	0.33	0.32	0.03	0.31	0.16	0.13

Ethanol and glycerol levels were similar for all the analyzed vinification products, and always fell within the standard range in wine, although ethanol production is generally higher after argan oil treatment, which is consistent with the improvement in fermentative capacity. However, acetic acid accumulation was clearly reduced for both pure culture vinifications using the argan-treated ADY, and this effect was particularly relevant for that conducted by control strain T73 ADY as the acetic acid level produced when the starter was obtained in standard molasses was much higher (0.14 g/L) than obtained in the argan oil-supplemented molasses (0.004 g/L). Interestingly, the mixed vinification, where the *H. osmophila* ADY came from the argan oil-supplemented molasses, maintained adequate acetic acid levels (0.18 g/L), similar to the value obtained in the pure culture T73 vinifications.

The profile of volatile compounds (Table 4) showed that argan oil supplementation in ADY production diminished the accumulation of undesirable ethyl acetate in the *H. osmophila* pure culture fermentation, and also in the mixed multi-starter fermentations. Although mixed multi-starter fermentations reached higher ethyl acetate levels than the T73 fermentation, these levels fell within the range of values to confer wine pleasant aromas as ethyl acetate provides desirable characteristics from 150-200 mg/L.

Regarding desirable higher alcohol acetate esters (Table 4), argan oil supplementation had very little effect on their production, and the positive desirable contribution of *H. osmophila* on acetate esters accumulation was not found in the mixed multistarter vinifications.

Accumulation of higher alcohols, such as isobutanol, isoamyl alcohol and 2-phenylethanol (Table 4), were increased by the argan oil supplementation in the pure fermentations, but not in the mixed fermentations. However, their basal levels under these conditions were higher than in the pure culture fermentations.

Finally, the fatty acid esters group, such as caproate, ethyl caprylate and ethyl caprate, was measured (Table 4). In general, argan oil treatment increased their levels in the pure cultures vinifications, but not in the mixed fermentation.

## DISCUSSION

In the last few years, several studies that have evaluated the use of controlled mixed fermentations, using *Saccharomyces* and different non-*Saccharomyces* wine yeast species 2,28,29, have concluded that such mixed fermentations are a feasible way to improve complexity and to enhance specific characteristics of wines 27. The consequent need for non-*Saccharomyces* ADY production has revealed new technological challenges as this industrial process has been historically designed and optimized for high biomass yields with *S. cerevisiae* strains. Negative effects on the viability and vitality of *S. cerevisiae* cells have been described 30, largely due to the oxidative damage of cellular components caused by ROS production 31. We recently described how wine *S. cerevisiae *strains with naturally high antioxidant defenses underwent less oxidative damage and displayed high fermentative capacity after dehydration, and we tested easily predictable markers for this biotechnologically relevant behavior 11. We also showed that molasses supplementation with pure antioxidant molecules (ascorbic, caffeic or oleic acids), or with food-grade argan oil, diminished the oxidative damage associated with ADY production through different molecular mechanisms 14.

This work aimed to characterize the physiological properties of five non-*Saccharomyces* oenological yeasts species during the ADY production process to obtain biochemical and cellular clues to their industrial performance. We analyzed their physiological and biochemical oxidative state after biomass propagation and dehydration, and tested the effects of antioxidants molasses supplementation for ADY production.

In general, the analyzed non-*Saccharomyces *yeasts display poor global performance after ADY production, a trait that can be related to some markers for deficient oxidative defense, such as a low intracellular GSH/GSSG ratio and low catalase activity induction, and also to low trehalose accumulation in fresh cells, and particularly in dry cells. However, the correlation found between antioxidant defenses and fitness after dehydration was not as clear as we previously described for *S. cerevisiae* wine strains 14, which suggests that these non-*Saccharomyces* species have additional physiological determinants. We found that *C. stellata* biomass yield was higher than the *S. cerevisiae* strain (T73), which could be because it is a largely aerobic and fructophilic yeast 32,33,34 that can consume both glucose and fructose efficiently to result in higher biomass yields, whereas low oxidative defenses would mainly affect it resistance to dehydration, which would explain its low fermentative capacity. The described fermentative metabolism of *T. delbrueckii* agreed with its observed high fermentative capacity, and even reached a low biomass yield due to the oxidative stress associated with industrial yeast propagation 8. Nonetheless in 2003, the first commercial release by Chr. Hansen (https://www.chr-hansen.com) of*T. delbrueckii* was yeast blends with *S. cerevisiae* and*K. thermotolerans*, and subsequently on its own and also by Laffort (https://www.laffort.com/es) and Lallemand Inc (http://www.lallemand.com two other *T. delbrueckii* strains are also available, which indicates the interest in carefully selected and tested non-*Saccharomyces* yeasts.

Ascorbic, caffeic and oleic acids supplementation improved oxidative response, but only increased the biomass yield in non-*Saccharomyces* species. It is known that ascorbic acid stimulates mitochondrial glycerol 3-phosphate dehydrogenase 35 to allow glycerol rerouting to glycolysis. Moreover, ascorbic acid has been shown to efficiently scavenge ROS, protect membrane lipids against peroxidation 36, increase low glutathione (GSH) levels 16 and regulate 1-Cys peroxiredoxin activity 15. Accordingly, our results revealed that ascorbic acid lowered the lipid peroxidation level, increased the GSH/GSSG ratio and trehalose content, and induced glutathione reductase (GR) activity, but did not protect protein against carbonylation. Therefore, the stimulation of glycolysis and reduction of oxidative damage would increase biomass yield.

Oleic and caffeic acids supplementation exhibited the same effects on biomass yield and oxidative response by reducing lipid peroxidation, but not protein carbonylation. Oleic acid supplementation has been used in wine yeast to mitigate oxidative stress during must fermentation because the lipid composition of cell membranes affects the activities of membrane-associated enzymes and transporters 21. The use of oleic acid and glucose co-substrates has been recently found to increase biomass production by reducing the Crabtree effect 37. Oleic acid would lead to an increase of biomass production by reducing ethanol production because of increased tricarboxylic acid cycle activity and, therefore, global respiratory capacity. This effect would enhance biomass yield, but not fermentative capacity, which agrees with our results. Moreover, it has been found that accumulation of fatty acids allows an adaptation to the endogenous oxidative stress associated with higher levels of reduced glutathione 38, which is also consistent with our results. Finally, caffeic acid has been linked with apoptosis and anticancer and antifungal activities 16 and, at low doses, diminishes lipid peroxidation and blocks ROS due to activation of the cell wall integrity (CWI) pathway 39,40 and GSH homeostasis regulation 41.

Based on antioxidant molecules supplementation, we conclude that low fermentative capacity is inherent for the *Candida*, *Pichia* and *Hanseniaspora* species and is not related mainly to oxidative stress. However, low biomass yield correlates with low oxidative defense in association with deficient GSH synthesis.

Nevertheless, as the use of pure chemicals could prove controversial in food industries, we propose food-grade argan oil supplementation during biomass propagation for wine ADY production. Argan oil contains high levels of linoleic and oleic acids, and is rich in polyphenols and tocopherols, which exhibit significant antioxidant activity 24. Minor compounds, such as sterols, carotenoids, caffeic acid, ascorbic acid, and squalene, contribute to its nutritional, dietetic and organoleptic value, and also to its preservative and health properties 25. In this case, argan oil improved the fermentative capacity in the *C. stellata* and *Hanseniaspora* species, but did not increase biomass yields. Moreover, it significantly reduced lipid peroxidation, increased the GSH/GSSG ratio, raised trehalose levels, and modulated the activity of enzymatic activities such as GR and catalase. The beneficial effect of argan oil on fermentative capacity could be also mediated by preventing membrane damage, increasing unsaturated fatty acids levels and elevating membrane permeability 42. Therefore, the low fermentative capacity of non-*Saccharomyces* yeasts, such as *Hanseniaspora *species, could be improved by modulating membrane permeability. Moreover, argan oil supplementation in molasses during ADY production improved yeast viability and reduced fermentation times in the natural must vinifications inoculated with both pure culture and mixed starters, and did not affect the organoleptic and sensorial properties of the resulting wine. However, the effects on volatile compounds levels were more diverse and further studies are still necessary to adjust the relationship between non-*Saccharomyces* and *Saccharomyces* species in multi-starter fermentations.

## MATERIALS AND METHODS

### Strains and cultivation conditions

Six wine yeast strains were studied: the well-known commercial *S. cerevisiae* strain T73, which was used as a control 43, and five non-*Saccharomyces* wine yeast species, which were classified into three groups: (1) largely aerobic yeasts: *Candida stellata* from the Yeast Genetic Stock Center (SC 5314) and *Pichia fermentans* from the Centraalbureau voor Schimmelcultures, Fungal and Yeast Collection (CBS 7435); (2) apiculate yeasts with low fermentative activity, *Hanseniaspora osmophila* (CECT 1474) and *Hanseniaspora guilliermondii *(CECT 11027) from the Spanish Type Culture Collection (CECT); (3) the highly efficient fermentative yeast *Torulaspora delbrueckii* from Lallemand Inc. (D91)*.*

Precultures and molasses for the biomass propagation and YPGF medium for the fermentative capacity assays were prepared as previously described 30.

For the antioxidants treatments, the molasses medium was supplemented with 50 µM ascorbic acid, 5 µM caffeic acid or 6 mg/mL oleic acid. Argan oil was added at the ratio of 1:100 (v/v) to provide a final concentration of 6 mg/mL oleic acid in molasses 14.

### Biomass dehydration and rehydration conditions

Dehydration and rehydration procedures at laboratory scale were previously tested and compared to industrial practices 10. Yeast biomass was separated from the molasses medium by centrifugation at 3000 rpm and subjected to several washing steps with cold distilled water. The concentrated biomass (500 mg) was spread on open Petri dishes and dehydrated in an air flux inside an oven at 30°C for 24 h to reach approximately 8% relative humidity, as determined by weight loss, and dried biomass was kept at room temperature. For rehydration, distilled water was used to resuspend the dried biomass at 37°C for 10 min under static conditions, followed by 10 min with shaking at 140 x g 10,28,44.

### Pure culture and mixed vinifications

For the vinification experiments with pure cultures, Tempranillo must (Bodegas J. Belda, Fontanars dels Alforins, 2013) was sterilized with 0.2% (v/v) dimethyl dicarbonate for 48 h at 4°C. Sterile bottles that contained 30 mL of must were inoculated with the rehydrated biomass of *S. cerevisiae* T73 or *H. osmophila*, previously obtained by growth in standard molasses (control) or argan oil-supplemented molasses (argan) to an OD_600_ of 0.1. These bottles were incubated at 28°C with gentle agitation (125 rpm) without aeration until complete sugar consumption.

For the vinification experiments with mixed starters, sterile bottles that contained 20 mL of natural must were simultaneously inoculated with *S. cerevisiae* T73 ADY from the control molasses and the argan oil-supplemented molasses, and *H. osmophila* from the argan oil-supplemented molasses. T73 and *H. osmophila* were inoculated at OD_600_ 0.1 and OD_600_ 1, respectively.

All the vinification experiments were performed in triplicates.

### Viability measurement

Appropriate dilutions of the monoculture vinifications, using the ADY from the control molasses and the argan oil-supplemented molasses, were grown on YPD plates for 24 h at 30°C and colony-forming units (CFU) were counted. Survival percentage was calculated by taking maximum day of growth as 100%.

### Fermentative capacity measurement

Fresh cells and dry cells were rehydrated and inoculated (10^7^ cells/mL) in YPGF medium, as described elsewhere 45. CO_2_ production was measured at 10-minute intervals for 6 h in a Fermograph (ATTO Corporation, Japan). Fermentative capacity was expressed as mL of CO_2_ produced per 10^7^ cells. Experiments were carried out in triplicates.

### Glutathione and intracellular trehalose determination

Extracts were obtained from 100 mg of cells, and were used for glutathione and trehalose determination, as previously described 10,11,46,47. The amount of glutathione was expressed as nmol per mg of cells. The amount of trehalose is given as µg of trehalose per mg of dry cell weight. Experiments were carried out in triplicates.

### Catalase and glutathione reductase activities

Extracts were obtained from 50 mg of cells and assayed spectrophotometrically as described by Jakubowski and colleagues 48 for catalase activity, and as described by Murshed and colleagues 49 for glutathione reductase activity (GR). Catalase activity was expressed as µmol of H_2_O_2_ min^-1^ mg of protein^-1^ (U/mg prot). GR activity was expressed as µmol of GSSG min^-1^ mg of protein^-1^ (U/mg prot).

### Protein carbonylation measurements 

The protein carbonylation in crude extracts was measured by dinitrophenilhydrazine (DNPH) derivatization and Western immunodetection of protein-bound 2,4-dinitrophenylhydrazones, as previously described 9,50. The anti-2,4-dinitrophenol antibody (Sigma) was used at the 1/3500 dilution and the secondary antibody (goat anti-rabbit HRP conjugated, Amersham) was used at the 1/5000 dilution. The signals in blots were visualized using Lumigen TMA-6 (Amersham), images were captured with the Las1000 software (FujiFilm) and protein carbonylation was measured by an image analysis using the QuantityOne software (BioRad).

### Lipid peroxidation measurements 

Lipid peroxidation quantification was carried out by running a reaction of thiobarbituric acid with the malondialdehyde (MDA) product of oxidized fatty acid breakage, as previously described 10. Lipid peroxidation was expressed as pmoles of MDA mg of cells^-1^.

### Reducing sugar, ethanol, acetate and glycerol measurements

Reducing sugars during fermentation were measured by a reaction to DNS (dinitro-3,5-salycilic acid) 51. Ethanol, acetate and glycerol were measured with the kits provided by r-Biopharm following the manufacturer’s instructions.

### Volatile compounds measurements

Gas chromatography allowed the final wine volatiles to be analyzed, which was performed in a capillary gas chromatograph, Hewlett-Packard model 5890 series II, controlled with a Science TRACE GC Ultra gas Chemstation 3365, equipped with a detector FID flame ionization and provided with a capillary column HP-INNOWax 30 m long, 0.25 mm internal diameter and 0.1 .mu.m thick phase. 1.5 mL of sample from the end of the fermentations was used, with 0.35 g of NaCl and 20 mL of 2-heptanone 0.005% (w/v) as the internal standard. The samples analyses were performed in triplicates.

### Statistical analysis

Sample averages were compared using a Student’s *t*-test. The samples denoted (a) were significantly different from those labeled (b) with a *p* < 0.05, and also differed from those denoted (c) with a *p* < 0.05. The samples labeled (ab) were not significantly different from (a) and (b), but significantly differed from (c). The samples denoted (*) were significantly different from one another.

A multivariate analysis (general linear model) assessed the effect of supplementation with different antioxidants and strains on the oxidative stress parameters (biomass propagation, fermentation capacity, lipid peroxidation, protein carbonylation, protective metabolites and enzymatic activities). The results were statistically compared by using 2-way ANOVA and the Tukey HSD *post hoc* tests. Statistical hypothesis tests were used to check the null hypotheses (α=.05) (SPSS v22.0; IBM SPSS Inc.). A PCA (Principal Component Analysis) was generated to visualize a 2D plot of the first two principal components, which revealed potential grouping patterns among supplementations or facilitated the recognition of outlier groups using the PAST 3.05 statistical software package 52.
